# Diagnostic Utility of Thoracoscopy in Recurrent Exudative Pleural Effusion: A Single-Arm Interventional Study

**DOI:** 10.7759/cureus.90541

**Published:** 2025-08-19

**Authors:** Ganesh C Mohapatra, Tapan Kalani, Suman K Jagaty, Saswat Subhankar

**Affiliations:** 1 Respiratory Medicine, Kalinga Institute of Medical Sciences, Bhubaneswar, IND; 2 Pulmonary Medicine, Kalinga Institute of Medical Sciences, Bhubaneswar, IND

**Keywords:** exudative effusion, granulomatous, malignant, pleural effusion, thoracoscopy

## Abstract

Background and objectives

Recurrent pleural effusion has always posed a challenge for definitive etiological diagnosis because it relies on nonspecific investigative methods like pleural fluid analysis.The basic nature of fluid, like transudate or exudate, can be differentiated by Light’s criteria. The common causes of exudative effusion are tuberculosis, malignancy, and pneumonia. The non-specific method of diagnosis mainly depends upon pleural fluid biochemical and cytological tests in order to decide an empirical treatment. The blind method of pleural biopsy by introducing a specialized pleural biopsy needle into the pleural cavity is also not promising for accurate histopathological study in order to reach a definitive etiological diagnosis in exudative pleural effusion patients. The aim and objectives of this study are to determine the role and efficacy of medical thoracoscopy for definitive diagnosis in patients having exudative pleural effusion.

Methods

The study was carried out by taking a sample size of 61 patients fulfilling the inclusion and exclusion criteria, having recurrent exudative pleural effusion as per Light's criteria, admitted in the pulmonary medicine department of Kalinga Institute of Medical Sciences over a period of two years (2023-2025). All cases were subjected to medical thoracoscopy in the designated procedural suite of this hospital after doing the required investigations. Multiple pleural biopsy samples were obtained in each patient and sent to the histopathology lab to obtain tissue diagnosis. All the data of the study were kept for critical analysis to reflect in the results and conclusion.

Results

The mean age of cases was 58 years with a male-female ratio of 1.25:1. Shortness of breath was the most common symptom (56 cases, 91.8%) followed by cough (47 cases, 77.04%). Hypertension was the most common comorbidity found among the cases (16 cases, 26.2%). The most common radiology finding was gross pleural effusion (54 cases, 88.5%), followed by mass lesion (16 cases, 26.2%), thickened pleura (16 cases, 26.2%), and nodules (nine cases, 14.8%). Pleural tissue biopsy revealed malignancy in 41 patients (67.2%). Out of which adenocarcinoma was the most common subtype (30 patients, 49.2%), followed by squamous cell carcinoma (eight patients, 13.1%), and was inconclusive in four patients. Post-thoracoscopic minor complications were reported in only 8 patients; 6 patients (9.8%) had subcutaneous emphysema, and two patients (3.3%) developed persistent air leak. Those were resolved with conservative treatment.

Conclusion

This study favored medical thoracoscopy as an ideal and effective tool to achieve a definitive diagnosis in recurrent exudative pleural effusion with very minimal and self-limited complications. Its role and efficacy are undisputed in all cases of recurrent exudative pleural effusion in order to achieve a highly promising diagnosis, so that an early treatment plan can be initiated accordingly.

## Introduction

Recurrent pleural effusion is a frequently encountered diagnostic dilemma in respiratory medicine. While no standard definition exists, the condition is typically characterized by fluid accumulation in the pleural space following initial treatment [1]. Pleural effusion is a very common disease encountered in respiratory medicine [2]. Effusions can be either transudative or exudative. Transudates commonly result from heart failure, liver cirrhosis, or nephrotic syndrome, whereas exudates are typically due to infections, malignancy, inflammation, or autoimmune disorders. Diagnostic limitations of pleural fluid analysis often led to reliance on indirect markers such as lymphocytosis or elevated adenosine deaminase (ADA) for suspected tuberculosis, or the presence of atypical/malignant cells for cancer, creating ambiguity in clinical decision-making [3].

Despite the availability of various diagnostic tests, accurately identifying the underlying cause of pleural effusions remains a significant challenge [4]. Many cases of tuberculous pleural effusion are diagnosed based on indirect indicators from pleural fluid analysis, such as lymphocytosis and elevated adenosine deaminase (ADA) levels [5]. Similarly, numerous pleural effusions with uncertain causes are labelled as malignant based on the detection of malignant, atypical, or suspicious cells in cytological examinations. These indirect diagnostic clues often leave treating physicians uncertain about the most appropriate and definitive course of management for these patients. Medical thoracoscopy offers a novel diagnostic method for the diagnosis of recurrent exudative pleural effusion by allowing a complete direct visualization of the pleura and obtaining target tissue for histopathological examination with minimal complications [6]. Medical thoracoscopy obviates the need for indirect methods of diagnosis, thus eliminating diagnostic uncertainty [7].

The aim and objective of this study is to evaluate the role, efficacy, and accuracy of medical thoracoscopy in establishing a definitive diagnosis in patients with recurrent exudative pleural effusion. This research is crucial for accurately diagnosing such cases across various scenarios, thereby facilitating appropriate treatment and reducing patient distress.

## Materials and methods

Study criteria

This single-arm interventional study was carried out in the Department of Respiratory Medicine at Kalinga Institute of Medical Sciences, Bhubaneswar, which is a tertiary care center, from June 2023 to May 2025. The study included patients aged 18 years or older who presented with recurrent exudative pleural effusion. Light’s criteria were applied to differentiate between exudative and transudative pleural fluid [3]. According to Light’s criteria, an effusion is classified as exudative if at least one of the following is present: (1) pleural fluid-to-serum protein ratio exceeding 0.5, (2) pleural fluid lactate dehydrogenase (LDH) level above 200 IU or more than two-thirds of the upper limit of normal per lab reference, or (3) pleural fluid-to-serum LDH ratio greater than 0.6.

Patients were excluded if they had transudative effusions, bleeding disorders, hemodynamic instability, bacteriologically confirmed tuberculosis (via sputum or pleural fluid), a previously diagnosed case of lung cancer, traumatic hemothorax, or tested positive for Hepatitis B surface antigen (HBsAg), Hepatitis C virus (HCV), or human immunodeficiency virus (HIV). Individuals who were unwilling to undergo medical thoracoscopy were also excluded. A total of 61 patients meeting the inclusion and exclusion criteria were enrolled. The study received approval from the Medical Research Committee at Kalinga Institute of Medical Sciences (KIMS), Bhubaneswar, with the approval number KIMS/SLRC/PG/2023/125.

Methodology

All patients with a history of recurrent pleural effusion admitted to the Department of Respiratory Medicine at KIMS Hospital, Bhubaneswar, underwent a thorough evaluation that included age, gender, presenting complaints, and physical examination findings. After an in-depth clinical assessment, they were subjected to routine diagnostic tests such as complete blood count, liver and kidney function tests, fasting and postprandial blood glucose levels, and screening for viral infections, including HIV, HBsAg, and HCV. Imaging modalities, including chest X-rays and thoracic CT scans, were performed for all patients. Chest ultrasonography was used to estimate the quantity and depth of pleural fluid and to determine the safest and most suitable site for aspiration [8]. Under ultrasound guidance, pleural fluid aspiration was performed in each case, and the obtained samples were sent to the Central Laboratory at KIMS for biochemical and cytological evaluation to differentiate between transudative and exudative effusions. Patients diagnosed with exudative pleural effusion (n=61) fulfilling inclusion and exclusion criteria subsequently underwent medical thoracoscopy using the Olympus LTF-160 semi-rigid thoracoscope, conducted in the hospital's dedicated thoracoscopy suite. During the procedure, pleural biopsies were obtained from multiple targeted sites of each patient. The biopsy specimens, placed in containers filled with normal saline and appropriately labelled, were sent to the histopathology department for detailed examination. All data of each patient, like demographic details, clinical presentation, associated comorbidities, and laboratory findings, were meticulously documented for further analysis.

Statistical analysis

The sample size was determined in consultation with the institutional statistician and approved by the ethics committee. Qualitative data were summarized using frequencies and percentages, while quantitative data were presented using the median and interquartile range (IQR). The Kruskal-Wallis test and Pearson's Chi-square test were applied to assess differences in quantitative and qualitative variables, respectively. Pearson’s correlation analysis was conducted to examine the relationships between various factors, with results expressed as correlation coefficients along with 95% confidence intervals. All statistical analyses were performed using R software (version 4.4.3, R Foundation for Statistical Computing, Vienna, Austria). A p-value of less than 0.05 was considered statistically significant.

## Results

Among the 61 patients included in the study, the mean age was 58.52 years with a standard deviation of 11.12 years. The cohort consisted of 34 males (55.7%) and 27 females (44.3%), resulting in a male-to-female ratio of 1.25:1. Of these, 35 patients (57.4%) were smokers, and 16 individuals (26.2%) had previously undergone anti-tubercular treatment (ATT). The most commonly observed comorbid conditions were hypertension in 16 patients (26.2%), diabetes mellitus in four patients (6.5%), and chronic kidney disease in two patients (3.3%). The most commonly reported symptom was shortness of breath, observed in 56 patients (91.8%), followed by cough in 47 patients (77.04%), chest pain in 28 (45.9%), fever in eight (13.1%), and weight loss in five patients (4.8%). Among comorbid conditions, hypertension was the most prevalent, while shortness of breath was the predominant presenting complaint (Table 1).

**Table 1:** Demographic and clinical parameters ATT: Anti-tubercular treatment.

Parameter	Value
Mean age (years)	58.52 ± 11.12
Age group	n (%)
≤ 50	12 (19.7%)
51-60	20 (32.8%)
61-70	20 (32.8%)
71-80	7 (11.5%)
>80	2 (3.3%)
Gender	
Male	34/61 (55.7%)
Female	27/61 (44.3%)
Smoking	
Yes	35 (57.4%)
No	26 (42.6%)
ATT Intake	
Yes	16 (26.2%)
No	45 (73.8%)
Co-morbidities	
HTN	16 (26.2%)
DM	4 (6.5%)
CKD	2 (3.3%)
Clinical Features	
SOB	56 (91.8%)
Cough	47 (77%)
Chest pain	28 (45.9%)
Fever	8 (13.1%)
Weight loss	5 (8.2%)

Radiological findings showed gross pleural effusion in 54 (88.5%) cases, mass lesions in 16 (26.2%) cases, pleural thickening in (26.2%) cases, nodules in nine (14.7%) cases, Hydropneumothorax six (9.8%) cases, mediastinal lymphadenopathy six (9.8%) cases, moderate pleural effusion three (4.9%) cases and consolidation three (4.9%) cases. Gross pleural effusion was the predominant finding (Table 2).

**Table 2:** Radiological characteristics of patients

Radiology	n (%)
Gross pleural effusion	54 (88.5%)
Mass lesion	16 (26.2%)
Thickened pleura	16 (26.2%)
Nodules	9 (14.7%)
Hydropneumothorax	6 (9.8%)
Mediastinal lymphadenopathy	6 (9.8%)
Moderate pleural effusion	3 (4.9%)
Consolidation	3 (4.9%)

Pleural fluid was straw-colored in 35 patients (57.4%) and hemorrhagic in 26 (42.6%). Cytological analysis revealed lymphocytic predominance in 47 cases (77%), neutrophilic cells in three (4.9%), malignant cells in nine (14.7%), atypical cells in one (1.6%), and mesothelial cells in one (1.6%). Lymphocytosis was the predominant finding in pleural fluid cytology (Table 3).

**Table 3:** Gross appearance and cytology of pleural fluid

Appearance	n (%)
Straw colored	35 (57.4%)
Hemorrhagic	26 (42.6%)
CYTOLOGY	
Lymphocytic	47 (77%)
Malignant cells	9 (14.7%)
Neutrophilic	3 (4.9%)
Atypical cells	1 (1.6%)
Mesothelial cells	1 (1.6%)

Biochemical analysis of pleural fluid showed the following mean values for each parameter like glucose 93.8 ± 46.1 mg/dL, protein 4.8 ± 0.85 g/dL, adenosine deaminase (ADA) 16.5 ± 11.3 IU/L, and lactate dehydrogenase (LDH) 781.7 ± 132.5 U/L (Table 4).

**Table 4:** Biochemical parameters of pleural fluid ADA: Adenosine deaminase, LDH: Lactate dehydrogenase.

Parameter	Mean ± SD
Glucose (ng/ml)	93.8 ± 46.1
Protein (g/dl)	4.8 ± 0.85
ADA (IU/l)	16.5 ±11.3
LDH (U/l)	781.7 ± 132.5

Thoracoscopic examination of the pleura revealed nodules in 49 (80.3%), ulceration in 27 (44.3%), adhesions in 23 (37.3%), and plaques in two (3.3%) cases (Table 5).

**Table 5:** Gross appearance on thoracoscopy

Gross appearance on thoracoscopy	n (%)
		Nodules	49 (80.3%)
Ulceration	27 (44.3%)		
		Adhesions	23 (37.3%)
Plaques	2 (3.3%)		

Biopsy findings of pleural tissue revealed malignancy in 41 patients (67.2%). Among the malignant cases, adenocarcinoma was the most prevalent subtype, identified in 30 patients (49.2%), followed by squamous cell carcinoma in eight cases (13.1%), adenosquamous carcinoma in two cases (3.3%), and metastatic clear cell carcinoma in one case (1.6%). Other findings included granulomatous inflammation in 10 patients (16.4%) and mesothelial hyperplasia in six patients (9.8%). In four cases (6.6%), the biopsy results were non-diagnostic (Table 6).

**Table 6:** Pleural tissue biopsy findings

Biopsy finding	n (%)
Malignancy	41 (67.2%)
Malignancy sub types	
Squamous cell carcinoma	8 (13.1%)
Adenocarcinoma	30 (49.2%)
Adeno-squamous carcinoma	2 (3.3%)
Metastatic clear cell carcinoma	1 (1.6%)
Others	
Granuloma	10 (16.4%)
Mesothelial hyperplasia	6 (9.8%)
Inconclusive	4 (6.6%)

Thoracoscopy-related minor complications were noted in eight patients (13.1%), with subcutaneous emphysema occurring in six (9.8%) cases and persistent air leak in 2 (3.3%) cases.

## Discussion

This single-arm interventional study analyzed 61 cases of recurrent exudative pleural effusion, selected based on defined inclusion and exclusion criteria, over a span of two years. The average patient age was 58.52 ± 11.2 years, ranging from 18 to 80 years, with a higher proportion of males. Shortness of breath emerged as the most common symptom. Dyspnea in pleural effusion is attributed to several factors, including impaired gas exchange, disrupted respiratory mechanics, and elevated intrapleural pressure [9]. The relatively high average age of the patients suggests a possible age-related predisposition to malignancies. The intensity of breathlessness typically reflects the extent of pleural effusion, ranging from gross to minimal, and the resulting pleural thickening, which limits chest wall expansion.

Thoracoscopy is a valuable tool for establishing a definitive diagnosis in cases of malignant pleural effusion. It allows direct visualization of the pleural cavity and enables targeted biopsy of abnormal pleural tissue. According to Wu et al., thoracoscopy can identify pleural malignancy in 92.6% of patients with previously undiagnosed pleural effusions, while 7.4% remain inconclusive [10]. In our study, pleural malignancy was diagnosed in 41 67.2% of cases, with four cases (6.6%) yielding inconclusive biopsy results. In addition to malignancy, we also identified other conditions, such as granulomatous inflammation in 10 (16.4%) and mesothelial hyperplasia in six (9.8%) cases. Wu et al. noted no recurrence of effusion in patients after 12 months of follow-up. Similar outcomes have been reported in other studies [11,12]. Malignancy continues to be recognized as the leading cause of recurrent pleural effusions of unknown origin [13-17].

Several earlier studies have identified pleural nodules as the most frequently observed thoracoscopic finding in cases of undiagnosed pleural effusion [18]. Consistent with these findings, our study also revealed pleural nodules as the most common abnormality, present in 49 (80.3%) cases. We observed a significant association between nodular lesions and malignancy (p = 0.0004). Our study found a strong association between gross pleural effusion on CT thorax and malignant pleural disease, which is consistent with earlier research [19]. Lymphocytosis was the predominant cellular pattern in pleural fluid, observed in 47 (77%) cases, echoing findings from previous studies [20,21]. Therefore, correlating pleural fluid cytology with clinical presentation and radiological features is essential in evaluating all cases of exudative pleural effusion.

Adenosine deaminase (ADA) levels are generally low in malignant pleural effusions, as these conditions typically do not trigger significant pleural inflammation, unlike tuberculous or parapneumonic effusions. Although mildly elevated ADA levels can occasionally be seen in malignant effusions, markedly higher levels are more characteristic of tubercular and parapneumonic cases [22]. Both low and high ADA levels in malignant pleural effusion have been linked to poorer prognosis [23].

Nevertheless, relying on low ADA levels alone to diagnose malignant pleural effusion is not appropriate, as there is no direct biochemical correlation supporting this. In our study, malignant effusion cases demonstrated lower mean ADA levels. Previous research has shown an inverse relationship between pleural fluid ADA levels and malignancy [24]. Patients with extensive malignant pleural effusion have been found to have significantly higher red blood cell (RBC) counts (p < 0.001) and lower ADA levels (p < 0.001). These two parameters were identified as independent predictors of malignancy using a stepwise logistic regression model [25]. In line with earlier studies, our findings revealed adenocarcinoma (30, 49.2%) as the most frequently occurring malignancy [26].

Additionally, our study emphasized the usefulness of medical thoracoscopy in identifying granulomatous diseases, which were found in 10 cases (16.4%). These granulomatous findings were suggestive of tuberculosis. However, no definitive evidence of tuberculosis was detected through histopathological analysis, Ziehl-Neelsen (Z-N) staining, or the Truenat test conducted on the biopsy specimens. Mesothelial hyperplasia was noted in 10 (9.8%) cases.

Medical thoracoscopy is considered a safe procedure when carried out by trained professionals, with a reported mortality rate of less than 1% [27]. A meta-analysis found no mortality associated with medical thoracoscopy, while the rates of major and minor complications were approximately 1.5% and 10.5%, respectively [28]. Similarly, in our study, no major complications were observed following thoracoscopy, further supporting its safety. Other studies have also reported minimal significant complications related to the procedure [29].

A limitation of this study was its small sample size, primarily because many patients were reluctant to provide consent for the invasive thoracoscopy procedure after it was explained to them. Additionally, there were challenges in maintaining follow-up with all patients after discharge. The study spanned two years, which may not be adequate to assess long-term outcomes, especially the possible recurrence of pleural effusion if the underlying condition progresses to a more chronic stage.

## Conclusions

Malignancy is the most commonly identified cause of recurrent exudative pleural effusion. Medical thoracoscopy is strongly recommended for all such patients to achieve a definitive diagnosis. This minimally invasive procedure enables direct inspection of the pleural cavity and allows for the precise collection of biopsy samples for histopathological analysis, as well as cultures for tuberculosis, fungal infections, and immunohistochemistry. It is considered a safe method with a very negligible complication rate. Our study also emphasizes the value of thoracoscopy in suspected cases of tubercular pleural effusion. Comprehensive clinical and radiological evaluation is crucial in each case, and thoracoscopy should be routinely employed in such patients unless contraindicated. This strategy can greatly reduce diagnostic delays and help initiate appropriate treatment promptly.
